# Use of equine pericardium sheet (LYOMESH®) as dura mater substitute in endoscopic endonasal transsphenoidal surgery

**Published:** 2013-09-02

**Authors:** Luigi M. Cavallo, Domenico Solari, Teresa Somma, Alberto Di Somma, Carmela Chiaramonte, Paolo Cappabianca

**Affiliations:** Division of Neurosurgery, Department of Neurosciences, Reproductive and Odontostomatological Sciences, Università degli Studi di Napoli Federico II Naples, Italy

**Keywords:** Endoscopic Endonasal Surgery, Skull Base Reconstruction, CSF leak, Dural Substitutes

## Abstract

**Objective:**

The aim of this study was to describe the use of equine pericardium sheet (Lyomesh ^®^ ) as dural substitute for sellar reconstruction after endoscopic endonasal transsphenoidal surgery for the removal of pituitary adenomas.

**Methods:**

We reviewed data of patients that underwent surgery by means of an endoscopic endonasal transsphenoidal approach for the removal of pituitary adenomas over a 12-months period, starting in May 2012, i.e. when we adopted Lyomesh ^®^ (Audio Technologies, Piacenza, Italy) an equine pericardium sheet, as dura mater substitute.

**Results:**

During the 12-months period evaluated, we performed an endoscopic endonasal transsphenoidal operation for a variety of pituitary lesions on 102 consecutive patients. Among these, in 12 patients (9.4%) harboring a pituitary adenoma, the implant of the pericardium sheet was used. Four patients (33.3%) presented a small intraoperative cerebrospinal fluid (CSF) leak; in these cases the Lyomesh ^®^ was placed intradurally with fibrin glue and, thereafter, several layers were positioned in extradural space. In 8 other subjects without any evidence of CSF leak, the dural substitute was placed intradurally and fibrin glue was injected intradurally to hold the material in place.

**Conclusions:**

Even if based on a relatively small patient series, our experience demonstrated that the use of equine pericardium sheet (Lyomesh ^®^ ) as dura mater substitute in transsphenoidal surgery is safe and biocompatible, as compared with other dural substitutes.

## INTRODUCTION

The widespread use of the endoscopic techniques has widened the possibilities of the transsphenoidal surgery, providing access to several areas of the skull base. Notwithstanding a consistent number of advantages, still there are several drawbacks, among those the postoperative CSF leakage. It has to be minded that this risk is different between so called “standard” procedures, targeted to the sellar area and the “extended” procedures, targeted mostly to the whole midline skull base. Indeed, in recent series reporting outcomes of the transsphenoidal approach, either endoscopic or microscopic, for the removal of sellar or intra-suprasellar infradiaphragmatic lesions, the rate of postoperative CSF leak is low, ranging from 0% to 5.1%; on the other side, in the so called “extended” procedures this rate is higher, ranging from 0% to 21% [ 1 – 12 ] . Several different prediction factors have been identified, such as elevated BMI (body mass index) that could directly correlate with the occurrence of CSF leakage [ 13 ] , although it stands clear that the ineffective repair of an intraoperative CSF fistula could be assumed as the major cause of such postoperative complications. Furthermore, the postoperative CSF leakage, if untreated, could create a direct communication between nasal cavities, and the brain, thus resulting in meningitis [ 2 , 6 , 14 , 15 ] . As well, the loss of CSF could lead to a tension pneumocephalus, representing another potentially devastating complication of this type of surgery, occurring with lower rates, usually lesser than 0.5% [ 16 – 18 ] .

Therefore, in recent years, different sellar and skull base reconstruction techniques have been described, accounting on an extreme variety of strategies and materials, i.e. autologous, heterologous and synthetic. There is not a univocal consensus concerning the best reconstruction option and strategies and materials should be tailored according to every single case.

The use of autologous materials (fat, muscle, fascia lata) might be preferred, because they are vital and do not engender any immune and/or inflammatory response. However, many of these substances require a separate surgical incision, either on the abdomen or on the thigh. With the modern minimally invasive conceptual way of thinking and the reduction of morbidity and mortality, the patients’ requests are leant toward the avoidance of a second skin incision. For these reasons, and for the possibilities offered by these products of reliable sterility, the use of non-autologous dural substitutes, both heterologous and synthetic, has received a tremendous boost. In this environment, pericardium-based heterologous products are becoming increasingly popular. Lyomesh ^®^ (Audio Technologies, Piacenza, Italy) is an equine pericardium membrane, used as dural substitute, recently introduced in the market; it is made of polypeptides chains, forming the elementary fibers, interwoven and shaped as cords, then anastomosed in complanate networks.

The aim of this study is to evaluate the safety and the effectiveness of this material for sellar reconstruction after a standard endoscopic endonasal approach for the removal of a pituitary adenoma.

## MATERIALS AND METHODS

### Patient population

We retrieved data from the analysis of 102 patients that underwent surgery for pituitary lesions, from May 2012 to May 2013.

The Lyomesh ^®^ dural foil was used in 12 cases harboring a pituitary adenoma: in four of them it was adopted to repair an intraoperative CSF leakage - grade I according to Kelly’s paradigm -, whereas in eight cases to protect a thinned suprasellar cistern - grade 0 according to Kelly’s paradigm [ 19 ] -.

We reported the use of such dural substitute in three PRL-secreting macroadenomas, two ACTH–secreting macroadenomas and seven non-functioning macroadenomas.

There were five males and seven females (mean age 52.5, ranging from 35 to 70 years). The postoperative follow-up ranged from 3 to 12 months.

The technique of reconstruction was different according to the entity of leakage to the anatomical and/or lesion features.

All patients underwent a three months postoperative endoscopic endonasal exploration of the sino-nasal cavities and a sellar MRI scan to evaluate the integration of Lyomesh ^®^ .

### Surgical technique

All patients underwent surgery by means of an endoscopic endonasal approach to sellar region for the removal of a pituitary adenoma, according to technique already described in the main literature [ 20 – 27 ] .

For the reconstruction phase of the standard procedure, we usually don’t harvest any autologous material such as abdominal fat graft or fascia lata, nor remove the middle turbinate to be used as free mucoperichondrium flap.

In case of small intraoperative CSF weeping leak (grade I) or also to protect a thinner and/or prolapsed suprasellar cistern (grade 0) [ 19 ] , we performed the repair of the osteodural defect by mean of different techniques after tumor removal. The Lyomesh ^®^ foil was placed intradurally in the eight cases presenting a CSF leak grade 0, when the suprasellar cistern was intact, prolapsed into the sellar cavity but thinner; fibrin glue was injected intradurally to hold the material in place.

In four cases with grade 1 intraoperative CSF leak, a single layer of Lyomesh ^®^ was placed intradurally with fibrin glue and, thereafter, several layers were positioned in the extradural space to ensure the watertighteness.

In two cases of this latter group the sphenoid sinus was filled with fibrin glue.

### Manufacture and handling of the collagen foil

Lyomesh ^®^ is a processed equine pericardium deproteinized with enzymatic method that preserves only purified collageneous network. The pericardium foil is made up of complanate multi-layer networks, rendered thinner by machinery process. Before its use the dural substitute must be washed at least 10 minutes in 50cc physiological saline solution. A full load of sheet measures is available.

## RESULTS

There were two main reasons that required the use of the dural substitute: the first one was an intraoperative CSF leak (grade I according to Kelly [ 19 ] - occurred in four cases -, while the second one was the prolapse of an intact, thinner suprasellar cistern after tumor removal - occurred in eight cases -.

**Fig 1:**
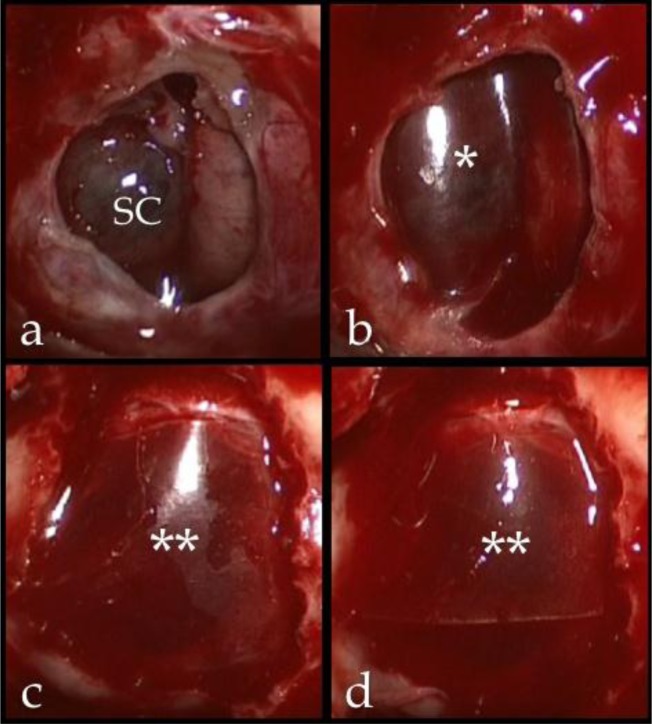
a: Intraoperative image showing a thinned suprasellar cistern descending into the sellar cavity after the removal of a pituitary adenoma; b: a pericardium sheet was used to protect and enforce the suprasellar cistern and prevent its postoperative rupture and consequent CSF leak; c, d: multiple layers of dural substitute placed in extradural space to close the sellar floor *: single layer of Lyomesh® placed intradurally, over the Suprasellar Cistern; **: Lyomesh® placed extradurally; SC: suprasellar cistern.

When intraoperative CSF leak was evident, the dural substitute was directly placed over the leaking points and intrasellar fibrin glue was injected [ 1 , 28 ] ; multiple layers of Lyomesh ^®^ were positioned in the extradural space to complete the repair of the osteodural defect ( Fig. 1 ).

**Fig 2:**
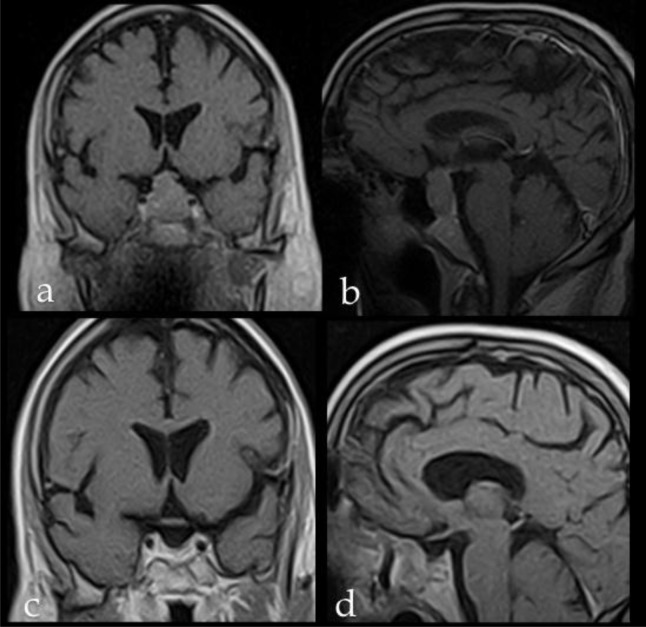
Preoperative and postoperative sellar MRi of a patient with an intra-suprasellar non-functioning pituitary adenoma, who received the implant of Lyomesh ^®^ . Preoprative (a) coronal and (b) sagittal scans. Postoperative (c) coronal and (d) sagittal scans showing no direct or indirect sign of complications related with the presence of Lyomesh ^®^ .

When there was not any evidence of CSF leak, the pericardium sheet was placed over the suprasellar cistern and then supported by the intrasellar injection of fibrin glue [ 1 , 28 ] . Three months MRI postoperative endoscopic control and sellar MRi showed the complete integration of the dural substitute ( Fig. 2 )

We did not observe any adverse clinical reactions directly related with the use of the dural substitute or any post-operative CSF leak (see Table I ).

## DISCUSSION

Traditional reconstruction techniques used in transcranial surgery, such as dural suturing and bone flap fixation, are not feasible after transsphenoidal surgery so that the osteodural reconstruction represents a main issue of this kind of surgery, often resulting troublesome. Sellar reconstruction after transsphenoidal pituitary tumor removal it is not mandatory in all cases but it is necessary in case of intra-operative CSF leakage or in presence of conditions that potentially expose to such event, such as the prolapse of suprasellar cistern into the sellar cavity [ 1 , 28 ] .

Although autologous materials (fat, muscle, fascia lata) might be preferred because of their biocompatibility and the lesser risk of immune or inflammatory response, they are harvested through a separate surgical incision, either on the abdomen or on the thigh that prolongs the surgical times and could be aesthetically disfiguring. On the other hand, it has to be said that, during endoscopic endonasal surgery, autologous materials could be harvested from the nasal structures i.e. middle turbinate and/or septal mucosa.

Heterologous materials, conversely, should be preferred to the synthetic ones because they are not absorbable and their inertness is still not absolute, with the possibility of causing a cell-mediated immunoallergic response [ 29 ] .

Lyomesh ^®^ has never been studied before for the use in this type of surgery. Thanks to the data collected along our series we have drawn some considerations in regards:

1. Watertightness: it provides a fluid-tight barrier against CSF leakage;
2. safety and biocompatibility: differently from bovine-derived dural substitute, this one is taken from horse, the only animal BSE safe, so there is no risk of infection transmission to human. It is biologically tested, non cytotoxic, non sensitizing, non mutageneous, with zero intracutaneous reactivity. The device is biocompatible and releases molecules that are metabolized without flogistic response. Nevertheless it should be used in patients allergic to horse meat;
3. easiness to handle: can be molded and shaped according to different needs of the repairs;
4. transparency: the transparency of Lyomesh ^®^ enables the surgeon to optimally inspect the underlying tissues;
5. mechanical resistance: the sheet traction and pressure resistance indexes are far higher than the one of the collagen tissues obtained through purification and artificial reticulation. Lyomesh ^®^ shows the mechanical resistance/thickness best ratio if compared with other biologic tissues of animal origin and to the artificial collageneous sheets;
6. slow resorption: it remains unchanged while fibroblasts and endotelial cells enter it, acting as a guide for connective regeneration while it is slowly digested and replaced by autologous connective tissue.

Since no previous studies are currently available to assess its security and effectiveness in transsphenoidal pituitary surgery, we used this new dural substitute only in cases of small CSF weeping, to protect the suprasellar cistern after the tumor removal or to close the sellar floor.

In fact, especially in cases of large or invasive adenomas, the suprasellar arachnoidal membrane could be thinned and the diaphragma sellae could be incompetent: even if there is not intraoperative evidence of CSF leak, this latter may occur later. Therefore, in these cases, the graft was kept in place by the sole fibrin glue that has proven in preliminary studies to be sufficient for this purpose [ 28 – 30 ] .

Due to the limited experience we would not yet recommend this material as sole substitute in case of large intraoperative CSF leak. In these cases, we followed the paradigm of graded sellar repair with multiple materials, combined to seal the different compartments of the defect [ 1 ] .

## CONCLUSION

Our preliminary results, even on a small patient series, demonstrate that the pericardium sheet, i.e. the Lyomesh ^®^ , presents advantages at least comparable with previous studies on similar materials [ 31 ] : it is safe, it is easy to handle and it is effective as dural substitute for the standard endoscopic procedures for pituitary adenomas removal.

## Figures and Tables

**TABLE I:** P atient population and sellar reconstruction technique .

** No. **	** Age/Sex **	** Adenoma type **	** CSF leak grade **	** Sellar reconstruction technique **	
				** Lyomesh ^ ® ^ position **	** Fibrin glue **
1	35, F	NF	0	Intradural space	sellar cavity
2	37, M	Prl-sec	1	Intradural/extradural space	sellar and sphenoid sinus cavities
3	40, F	Acth-sec	0	Intradural space	sellar cavity
4	45, M	NF	0	Intradural space	sellar cavity
5	47, F	Prl-sec	1	Intradural/extradural space	sellar cavity
6	50, F	Acth-sec	0	Intradural space	sellar cavity
7	55, M	Prl-sec	0	Intradural space	sellar cavity
8	58, F	NF	1	Intradural/extradural space	sellar and sphedoid sinus cavities
9	60, F	NF	1	Intradural/extradural space	sellar cavity
10	65, M	NF	0	Intradural space	sellar cavity
11	68, F	NF	0	Intradural space	sellar cavity
12	70, M	NF	0	Intradural space	sellar cavity
